# Recurrence of B-cell Lymphoma Manifesting as Progressive Peripheral and Cranial Nerve Neuropathies: A Case Report

**DOI:** 10.7759/cureus.115365

**Published:** 2026-08-28

**Authors:** Stan Schofield, Gurpreet Sehmbi, Shwe Tun, Rosaria Buccoliero

**Affiliations:** 1 Department of Neurology, Harrogate and District NHS Foundation Trust, Harrogate, GBR

**Keywords:** fdg-pet imaging, nerve biopsy, neurolymphomatosis, non-hodgkin lymphoma, zanubrutinib

## Abstract

Neurolymphomatosis (NL) is a rare neurological complication of lymphoma, characterised by direct infiltration of peripheral nerves by neoplastic cells. It most commonly occurs in association with non-Hodgkin’s lymphoma (NHL) and poses significant diagnostic challenges due to its non-specific clinical presentation. Classically, NL presents as a painful, rapidly progressive, and asymmetric neuropathy, frequently mimicking inflammatory or vasculitic neuropathies. Here, we present the case of a 69-year-old woman with a history of NHL, in remission for five years, who presented with a progressive, asymmetric sensorimotor polyneuropathy that later progressed to include several cranial nerve palsies. Initial investigations demonstrated features of chronic inflammatory axonal polyneuropathy. The patient’s atypical clinical progression and poor response to immunosuppressive therapy prompted further evaluation. A CT-guided sciatic nerve biopsy, based on fluorodeoxyglucose-positron emission tomography (FDG-PET) imaging, facilitated the histopathological analysis that confirmed infiltration by malignant B lymphocytes, establishing a diagnosis of secondary NL. Targeted therapy with zanubrutinib led to marked neurological improvement, although she ultimately passed away. This case highlights the importance of maintaining a high index of suspicion for NL in patients with atypical or refractory neuropathies and a history of lymphoma and underscores the diagnostic value of FDG-PET imaging and nerve biopsy in guiding accurate diagnosis and delivery of crucial early intervention.

## Introduction

Neurolymphomatosis (NL) is a rare disease characterised by the direct infiltration of peripheral nerves by neoplastic cells, most commonly associated with non-Hodgkin’s lymphoma (NHL) [1,2]. Clinically, NL typically presents as a painful, rapidly progressive, and often asymmetric neuropathy, frequently mimicking inflammatory or vasculitic neuropathies. NL can present in active or relapsed stages of disease [1]. The prevalence of NL has increased, and trends reported in a 2010 analysis indicate that post-mortem diagnoses have declined substantially since the millennium [1]. This shift can be attributed to greater disease awareness and, more importantly, advancement in contemporary diagnostic techniques. Despite this, an early diagnosis of NL is seldom made.

Here, we present a diagnostically and therapeutically challenging case of a woman with recurrent NHL presenting with a progressive asymmetric sensorimotor polyneuropathy. In contrast to classical presentations, her disease followed a more insidious course, with lymphoma being quiescent for several years. Furthermore, her clinical presentation initially suggested lumbar radiculopathy and subsequently demonstrated electrodiagnostic features consistent with chronic idiopathic axonal polyneuropathy (CIAP). Fluorodeoxyglucose-positron emission tomography (FDG-PET) and sciatic nerve biopsy were crucial in establishing the specific subtype of secondary NL, enabling initiation of zanubrutinib as a targeted therapeutic approach. This led to initial marked clinical improvement, although subsequent deterioration occurred several months later. This case emphasises the critical importance of ascertaining an early diagnosis, as prompt initiation of disease-directed therapy can improve the depth and durability of clinical response; however, the overall prognosis in secondary NL remains poorly defined.

## Case presentation

A previously independent 69-year-old woman presented with a six-month history of falls, left lower limb weakness, left thigh hypoesthesia, and gradual decline in mobility. Her past medical history included marginal zone lymphoma (MZL) diagnosed 10 years previously, treated with multiple chemotherapy regimens comprising rituximab, bendamustine, and corticosteroids, with sustained remission for five years before this presentation. Additional comorbidities included diet-controlled type 2 diabetes mellitus, vitamin D deficiency, and hypothyroidism. Neurological examination revealed dragging of the left leg during gait assessment, proximal weakness in the left lower limb, and areflexia in the lower limbs with no sensory deficit. The rest of the examination was normal.

The initial nerve conduction study (NCS) and electromyography (EMG) showed possible left L2/L3 root radiculopathy. However, study quality and interpretation were limited due to the patient’s body habitus. Subsequent whole-spine MRI scan did not reveal abnormalities at those levels. This was followed by a non-contrast MRI of the brain, which excluded vascular causes of the clinical presentation. Over the course of six months, the patient reported worsening mobility, including new bilateral foot weakness, right upper limb weakness, and pain in the lower limbs and buttocks. The repeated neurological examination demonstrated bilateral foot drop, proximal weakness of the left lower limb, weakness of the right triceps muscle, and weakness of right wrist extension and flexion, as well as all movements of the right hand. Tendon reflexes were absent in the upper and lower limbs, with impaired vibration and position sense in the right great toe.

**Figure 1:**
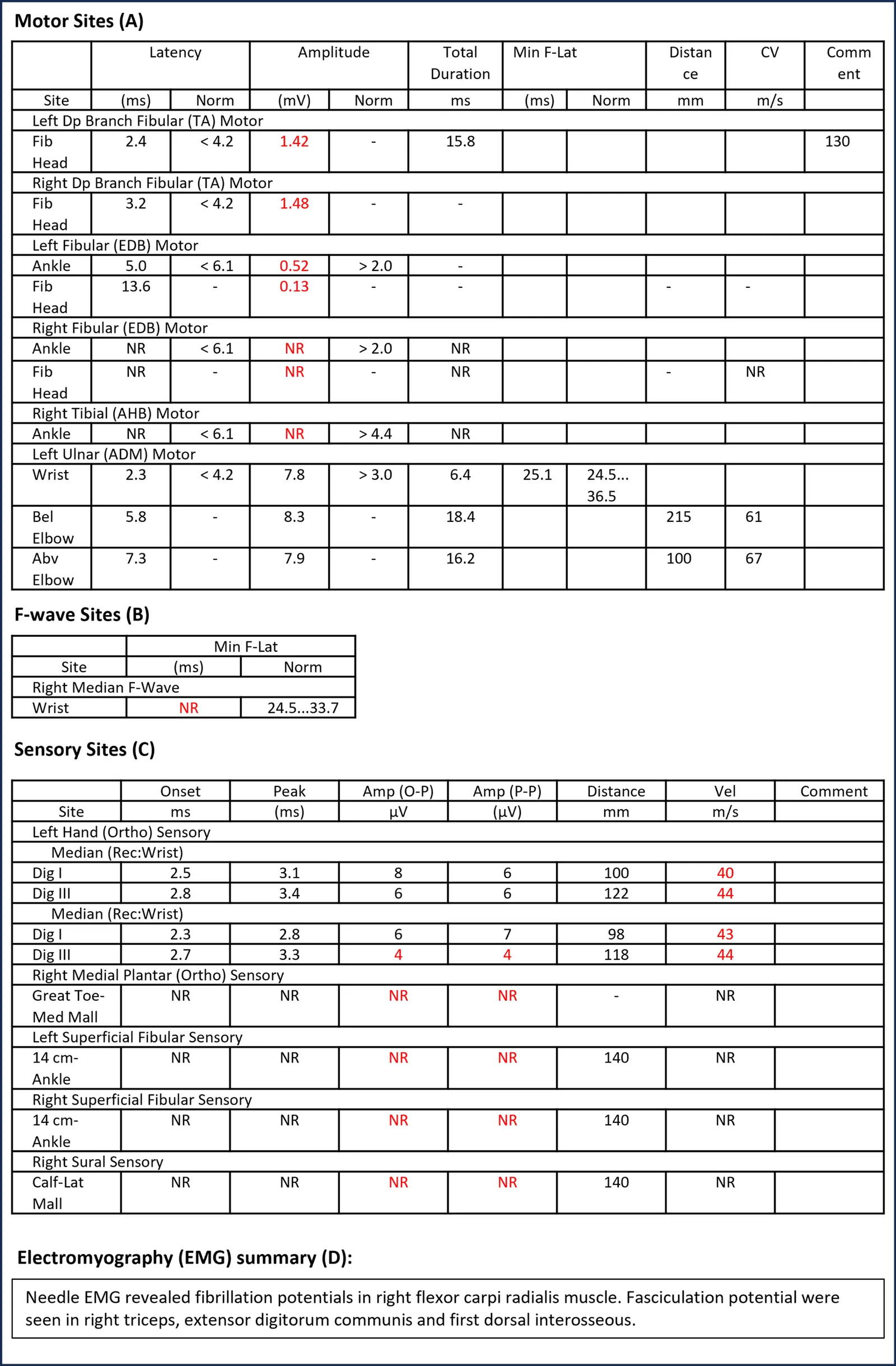
Repeated nerve conduction study and electromyography findings. (A) Motor nerve conduction study results. (B) F-wave study results. (C) Sensory nerve conduction study results. (D) Summary of electromyography findings. Red text indicates abnormal findings.

Following this, a repeated NCS/EMG revealed an asymmetrical generalised axonal sensorimotor polyneuropathy (poly-radiculo-neuropathy), suspicious of CIAP (Figure 1).

Oral corticosteroid treatment was initiated to treat a possible immune-mediated or vasculitic process as a differential for the NCS/EMG findings. Shortly thereafter, she experienced further deterioration and functional impairment, becoming wheelchair bound, necessitating admission for neurorehabilitation. Extensive laboratory investigations, including urine protein electrophoresis, serum immunoglobulins, cryoglobulins, hepatitis serology, infectious disease screening, endocrine and nutritional investigations (thyroid-stimulating hormone, vitamin D, parathyroid hormone, folate, and B12), and paraneoplastic and autoimmune screening (including anti-Ro60, anti-Ro52, anti-La, anti-Sm, anti-Sm/RNP, anti-RNP68, anti-Scl70, anti-Jo-1, anti-centromere, anti-chromatin, anti-ribosomal P, anti-myeloperoxidase, and anti-proteinase 3 antibodies) were unremarkable (Table 1). The initial cerebrospinal fluid (CSF) analysis revealed protein 0.98 g/L (0.2-0.4 g/L), pleocytosis 12 × 10⁶ cells/L (<5 × 10⁶ cells/L), and presence of atypical cells on flow cytometry, which was not diagnostic due to poor cellularity (Table 1). CT of the thorax, abdomen, and pelvis reported no recurrence of lymphoma.

**Table 1:** Summary of laboratory, cerebrospinal fluid, imaging, and ancillary findings with corresponding reference ranges and clinical interpretation. CSF = cerebrospinal fluid; WCC = white cell count; PCR = polymerase chain reaction; ACE = angiotensin-converting enzyme

Investigation	Parameter	Result	Unit	Reference	Interpretation
First CSF sample	Protein	0.98	g/L	0.20–0.40	Mild elevation
	WCC	12 × 10^6^	cells/L	<5 × 10^6^	Mild pleocytosis
	Glucose	3.2	mmol/L	>60% of serum	Reduced (serum glucose 6.6 mmol/L)
	Gram stain	No organism seen	-	Negative	No bacteria identified
	Culture	No growth	-	Negative	No bacterial growth
	Viral PCR	No virus detected	-	Negative	No virus detected
Repeated CSF sample	Protein	3.0	g/L	0.20–0.40	Elevated
	WCC	165 x 10^6^	cells/L	<5 x 10^6^	Pleocytosis
	Glucose	0.73	mmol/L	>60% of serum	Reduced (serum glucose 4.9 mmol/L)
	Gram stain	No organism seen	-	Negative	No bacteria identified
	Culture	No growth	-	Negative	No bacterial growth
Myeloma screen	Urine electrophoresis	No Bence Jones protein	-	Negative	Normal
	Serum electrophoresis	No paraprotein detected	-	Negative	Normal
Autoimmune screen	Anti-nuclear antibody	Negative	-	Negative	Normal
Paraneoplastic screen		Negative	-	Negative	Normal
HIV		Negative	-	Negative	Normal
Syphilis IgG/IgM		Negative	-	Negative	Normal
Hepatitis B surface antigen		Negative	-	Negative	Normal
Hepatitis B core antibody		Negative	-	Negative	Normal
Hepatitis C antibody		Negative	-	Negative	Normal
Lyme disease IgM		Negative	-	Negative	Normal
Lyme disease IgG		Negative	-	Negative	Normal
Cryoglobulin		Not detected	-	Negative	Normal
Serum ACE		<10	U/L	20–70	Normal
MRI of the spine		No nerve root/cord compromise	-	-	No explanation for clinical symptoms
MRI of the head (non-contrast)		Old tiny lacunar infarct involving right cerebellum. Mild microvascular disease	-	-	No explanation for clinical symptoms
MRI of the brain with contrast		Abnormal soft tissue within the left Meckel’s cave with thickening and enhancement of the proximal V3 nerve, and subtle bilateral facial nerve enhancement	-	-	Abnormal results

Unfortunately, the steroid course had minimal effect and was weaned. During steroid weaning, the patient developed left facial and abducens nerve palsies, progressing to bilateral facial nerve palsy within three weeks, followed by the development of a right oculomotor nerve palsy. Repeat MRI of the brain with contrast revealed subtle enhancement of the facial nerves and of the proximal mandibular division of the trigeminal nerve, suspicious of recurring lymphoma. Repeat CSF analysis showed markedly increased white cells 165 x 10⁶ (100% lymphocytes- CD5+ B cells) and protein 3.0 g/L (0.20-0.40 g/L) (Table 1).

**Figure 2:**
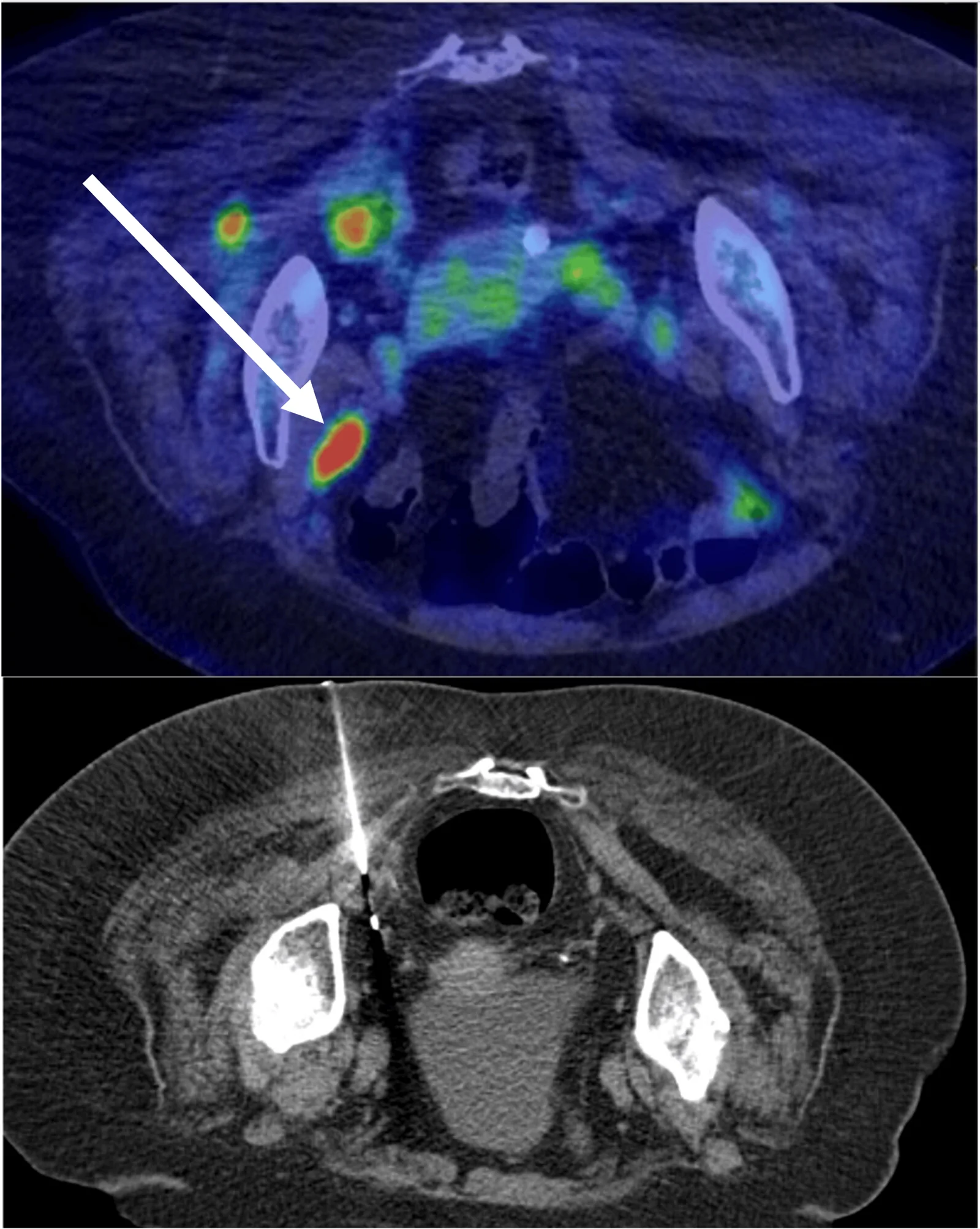
Above: FDG PET-CT slice at the level of the greater sciatic notch, demonstrating radiotracer avid uptake involving the right sciatic nerve (arrow). Below: CT-guided biopsy of the right sciatic nerve. FDG PET-CT = fluorodeoxyglucose positron emission tomography-computed tomography

CSF flow cytometry demonstrated abnormal lymphocytes; however, this was not confirmatory of the lymphoma type, as a fluorescence in situ hybridisation test could not be performed due to an insufficient sample. FDG-PET CT scan demonstrated multifocal, highly FDG-avid neural thickening which, in the clinical context, was suspicious for NL (Figure 2).

**Figure 3:**
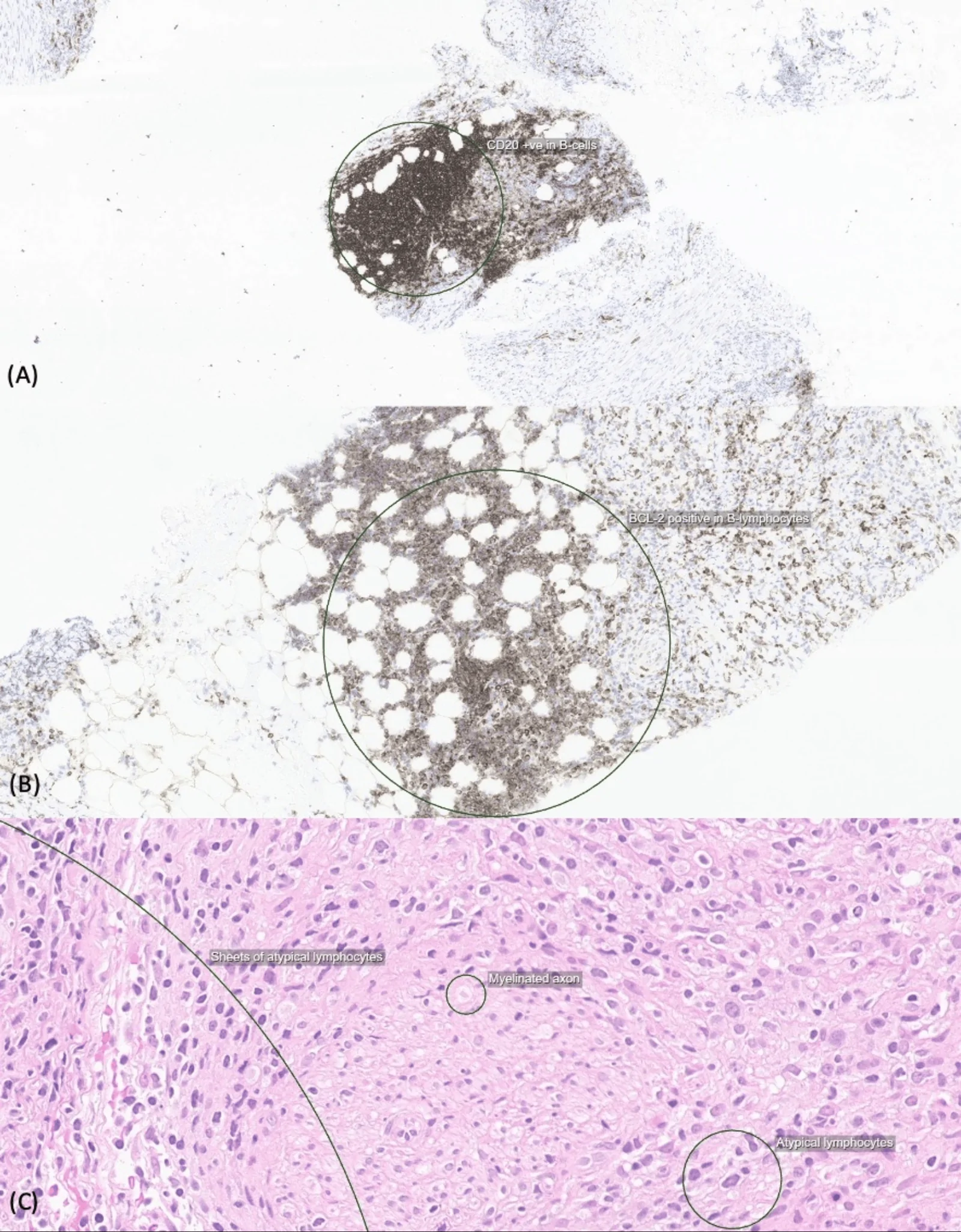
Histology slides demonstrating microscopic examination of the right sciatic nerve biopsy. (A) CD20 stain indicating infiltration by B lymphocytes (magnification ×20). (B) BCL-2 stain indicating neoplastic B lymphocytes (magnification ×20). (C) Haematoxylin and eosin staining indicating presence of atypical lymphocytes alongside myelinated axon (magnification ×5).

Additional differentials included neurosarcoidosis and infiltrative or inflammatory neuronal conditions, such as IgG4-related disease. Tissue biopsy was pertinent to confirm specifics of this diagnosis. The FDG-PET CT scan highlighted two possible biopsy sites as the right sublingual space and right sciatic nerve root, with the latter being identified as the most viable following a discussion with interventional radiology and an assessment from a maxillofacial surgeon. Histopathology testing from a CT-guided right sciatic nerve biopsy then confirmed a *MYD88*-mutated B-cell lymphoproliferative disorder (Figures 3A-3C).

Treatment with prednisolone and zanubrutinib, a Bruton’s tyrosine kinase (BTK) inhibitor, was commenced, resulting in substantial neurological improvement over the subsequent months. One month after initiating therapy, the right third cranial nerve palsy had fully resolved, with significant improvement in the left sixth cranial nerve palsy. By three months, she had regained the ability to stand independently, demonstrated improved proximal lower limb strength, and regained active left ankle dorsiflexion and toe flexion. Additionally, the right upper limb weakness had completely resolved, with near-complete recovery of the left sixth cranial nerve palsy. Despite this clinically meaningful neurological response, she subsequently deteriorated after five months with the development of hydrocephalus, consistent with advancing NL. Her condition was ultimately complicated by aspiration pneumonia, which was recorded as the cause of death.

**Figure 4:**
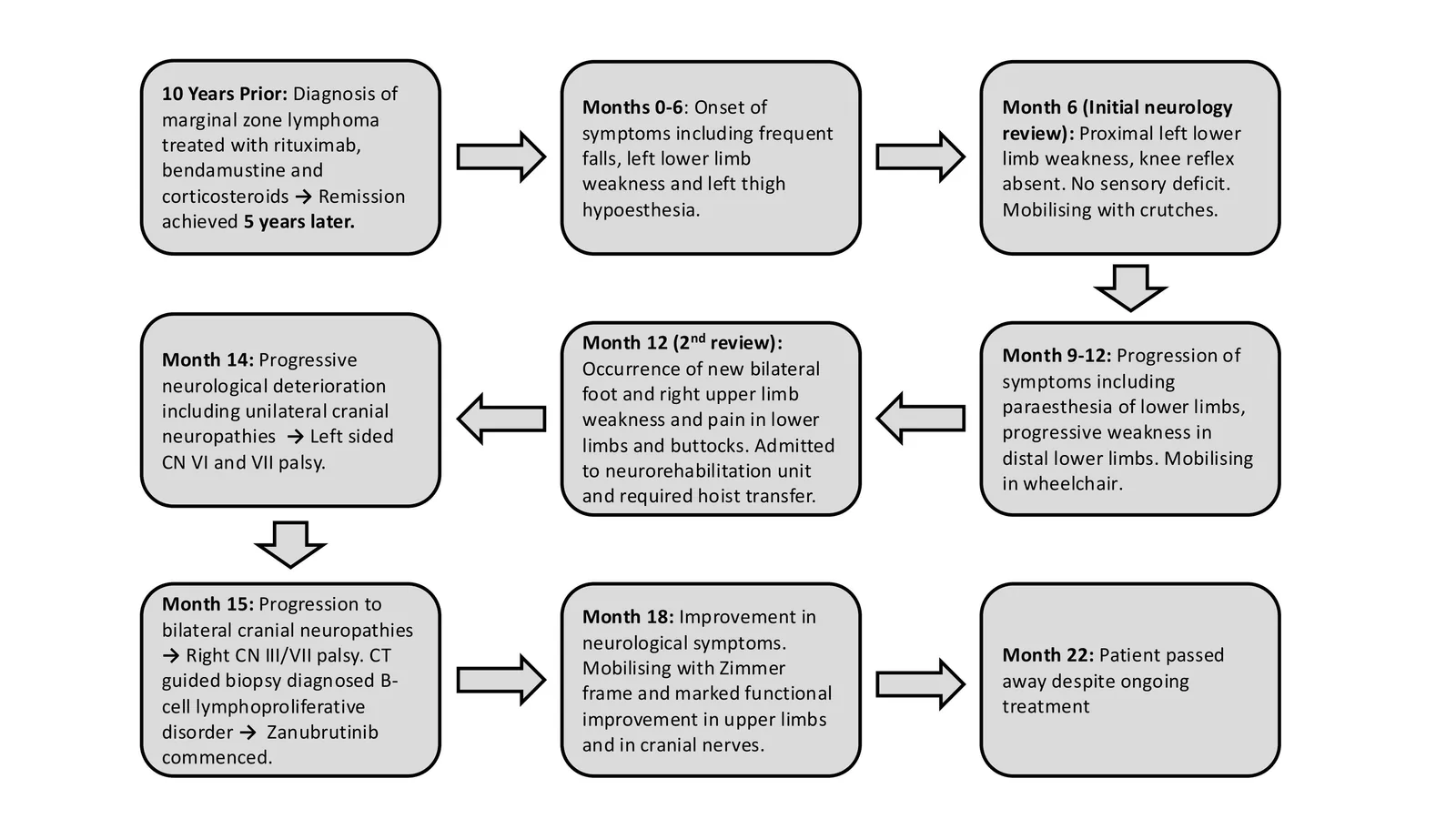
Chronological overview of clinical events. A summary of disease progression, including key clinical investigations and management.

A chronological timeline of events, to facilitate the understanding of this clinical case, starting 10 years before the onset of the patient’s neurological symptoms, is provided in Figure 4.

## Discussion

NL is a rare manifestation of lymphoma characterised by infiltration of the peripheral nervous system, with diagnosis often delayed due to its heterogeneous clinical presentation [1]. Historical estimates suggest a prevalence of approximately 0.2-0.5%, although this likely underestimates the true incidence due to under-recognition [3].

This case underscores the diagnostic challenge posed by NL, particularly in this presentation with a progressive asymmetrical sensorimotor polyneuropathy manifestation in a patient with a prior history of NHL. Timely initiation of treatment is often challenging, with delays frequently occurring between symptom onset and definitive diagnosis [4]. Notably, presentations of secondary NL are associated with worse outcomes compared to primary NL [1,5], highlighting the clinical necessity for prompt diagnosis and treatment.

NL typically presents as a sensorimotor neuropathy and affects multiple anatomical regions [1]. In a recent comparative study, mononeuropathies were observed more frequently in secondary NL than in primary NL (34% vs. 3%, p = 0.01) [5]. Clinically, NL can mimic non-paraneoplastic neuropathies such as inflammatory processes. However, the rapid progression and multifocal distribution of a painful neuropathy in the context of NHL in remission is clinically concerning for NL, even when the electrodiagnostic findings resemble those seen in CIAP [1]. In this case, the presence of painful and rapidly evolving neuropathies, including bilateral multiple cranial nerve palsies, raised concern for an underlying infiltrative or malignant process and prompted repeat investigations of CSF analysis and MRI of the brain. Bilateral facial nerve involvement is considerably rarer than unilateral involvement, necessitating urgent neuroimaging [6].

FDG-PET is well recognised to offer a high diagnostic yield for NL [1,7]. Although MRI of the head demonstrated thickening of affected cranial nerves, this was not specific to NL as it can be observed in inflammatory polyneuropathies [3]. In the context of strong clinical suspicion of NL, MRI findings may support suspicion for NL but cannot yield a definitive diagnosis alone; therefore, FDG-PET remains a superior modality of imaging to streamline further diagnostic interventions.

CSF analysis has demonstrated limited utility in NL diagnosis [1,8]. Despite NL being considered in the initial differential diagnoses, the first CSF analysis and CT of the thorax, abdomen, and pelvis did not demonstrate evidence of lymphoma recurrence. These findings, alongside features of chronic asymmetrical polyneuropathy and electrophysiological evidence of axonal neuropathy, instead supported an alternative diagnosis of inflammatory axonal polyneuropathy [9]. This justified an empirical trial of immunosuppressive therapy.

The diagnostic framework for CIAP has limited specificity in excluding infiltrative or neoplastic neuropathies, particularly as more common causes of neuropathy have overlapping clinical presentations. Features such as rapid progression of symptoms, painful neuropathy, and poor or transient response to steroid therapy can be considered diagnostic indicators of serious pathology and should prompt re-evaluation of the diagnosis to consider alternative aetiologies, including NL [10]. In these cases, a combination of MRI, FDG-PET imaging, and targeted nerve biopsy can help avoid fatal delays in diagnosis and treatment, as well as inappropriate use of immunosuppression.

In this case, the development of cranial neuropathies despite immunosuppressive therapy represented a significant red flag against the diagnosis of inflammatory axonal polyneuropathy. Repeat cytology and flow cytometry of CSF demonstrated significant lymphocytosis, hyperproteinaemia, and a new presence of neoplastic B cells. This temporal correlation underscores the value of repeat CSF sampling in refractory clinical presentations, as here it strongly supported the suspicion for NL, providing the basis for FDG-PET imaging and diagnostic biopsy thereafter.

Nerve biopsy carries a high diagnostic yield for NL [6]. It is also instrumental in confirming uncertain diagnoses, particularly given the heterogeneity of lymphoma subtypes requiring accurate classification to guide disease-specific treatment. Given the associated risk of permanent complications, there remains trepidation regarding its utility among clinicians [11]. Consequently, biopsy is typically reserved for patients with rapidly progressing symptoms or diagnostic uncertainty. The CT-guided sciatic nerve biopsy was well tolerated, with no reported complications, and was essential for definitive diagnosis and guiding targeted treatment. This highlights the importance of image-guided biopsy to improve accessibility and identify safe targets with higher diagnostic yield. In cases where nerve biopsy is not feasible, treatment should be considered based on the most likely diagnosis, with clinical response monitored accordingly.

Treatment strategies for NL are patient-specific and guided by the underlying lymphoma subtype. Although no consensus treatment has been established, B-cell lymphoma-associated NL is generally treated with systemic chemotherapy, including methotrexate or rituximab-based regimens [7]. Prognosis remains guarded, with a recent systematic review reporting a median survival of 18 months, with secondary NL carrying an inferior prognosis than primary NL [7]. Zanubrutinib is a second-generation BTK inhibitor that irreversibly inhibits B-cell receptor signalling, thereby reducing malignant B-cell proliferation and survival. Zanubrutinib is an established treatment for relapsed/refractory B-cell malignancies, with the MAGNOLIA study demonstrating efficacy in relapsed/refractory MZL and exploratory data suggesting favourable outcomes in *MYD88*-mutated disease [12]. Furthermore, zanubrutinib achieves central nervous system penetration, with emerging evidence supporting further investigation of BTK inhibitors for B-cell lymphoproliferative disorders involving the central nervous system [13].

Although literature supporting BTK inhibition specifically in B-cell lymphoma neuropathies remains limited, zanubrutinib was selected in this case due to relapsed *MYD88*-mutated B-cell lymphoma, the mechanistic rationale for BTK inhibition in *MYD88*-mutated disease, and its favourable central nervous system activity profile [14]. The patient demonstrated initial neurological improvement, including recovery of cranial nerve deficits and improved limb function; however, her disease subsequently progressed despite ongoing therapy. Her clinical course, ultimately complicated by aspiration pneumonia resulting in death, reflects the aggressive nature of relapsed NL and the limited evidence regarding the durability of these responses outside of the initial treatment phase [14].

## Conclusions

NL should always be considered in the differential diagnosis for patients with active, relapsed, or previously treated NHL presenting with rapidly progressive, asymmetric neuropathies, particularly in the presence of concerning features such as rapidly evolving bilateral cranial nerve involvement. In this case, the diagnosis became apparent only after the patient developed multiple cranial nerve palsies, with neuroimaging findings providing supportive evidence for NL. Accurate diagnosis relies on a combination of investigative modalities. This case epitomises the utility of FDG-PET and image-guided nerve biopsy in confirming a specific diagnosis of NL, which is essential to allow for timely targeted treatment. In this case, zanubrutinib resulted in an initial positive clinical response, highlighting the potential role of central nervous system-penetrating BTK inhibitors in selected patients with NL, particularly where methotrexate-based treatment regimens may be unsuitable. However, further evidence is required to clarify their efficacy and durability of response in secondary NL. Prompt initiation of central nervous system-penetrant therapy can lead to significant functional improvement, though the prognosis remains guarded in secondary NL.

## References

[1] Grisariu S, Avni B, Batchelor TT (2010). Neurolymphomatosis: an International Primary CNS Lymphoma Collaborative Group report. Blood.

[2] Bourque PR, Sampaio ML, Warman-Chardon J, Samaan S, Torres C (2019). Neurolymphomatosis of the lumbosacral plexus and its branches: case series and literature review. BMC Cancer.

[3] Baehring JM, Damek D, Martin EC, Betensky RA, Hochberg FH (2003). Neurolymphomatosis. Neuro Oncol.

[4] Pellegatta S, Poliani PL, Stucchi E (2010). Intra-tumoral dendritic cells increase efficacy of peripheral vaccination by modulation of glioma microenvironment. Neuro Oncol.

[5] Skolka MP, Suanprasert N, Martinez-Thompson JM (2024). Neurologic clinical, electrophysiologic, and pathologic characteristics of primary vs secondary neurolymphomatosis. Neurology.

[6] Teller DC, Murphy TP (1992). Bilateral facial paralysis: a case presentation and literature review. J Otolaryngol.

[7] Kaulen LD, Hielscher T, Doubrovinskaia S (2024). Clinical presentation, management, and outcome in neurolymphomatosis: a systematic review. Neurology.

[8] Byun JM, Kim KH, Kim M (2017). Diagnosis of secondary peripheral neurolymphomatosis: a multi-center experience. Leuk Lymphoma.

[9] Oh SJ, Lu L, Alsharabati M, Morgan MB, King P (2020). Chronic inflammatory axonal polyneuropathy. J Neurol Neurosurg Psychiatry.

[10] Makranz C, Arkadir D, Nachmias B (2021). Neurological misdiagnoses of lymphoma. Neurol Sci.

[11] Nathani D, Spies J, Barnett MH, Pollard J, Wang MX, Sommer C, Kiernan MC (2021). Nerve biopsy: current indications and decision tools. Muscle Nerve.

[12] Opat S, Tedeschi A, Hu B (2023). Safety and efficacy of zanubrutinib in relapsed/refractory marginal zone lymphoma: final analysis of the MAGNOLIA study. Blood Adv.

[13] Zhang Y, Li Y, Zhuang Z (2021). Preliminary evaluation of zanubrutinib-containing regimens in DLBCL and the cerebrospinal fluid distribution of zanubrutinib: a 13-case series. Front Oncol.

[14] Xing Y, Zhao K, Zhang Y, Wang Y (2024). BTK inhibition in primary central nervous system lymphoma: mechanisms, clinical efficacy, and future perspectives. Front Oncol.

